# Technologies for Opioid Use Disorder Management: Mobile App Search and Scoping Review

**DOI:** 10.2196/15752

**Published:** 2020-06-05

**Authors:** Joseph Nuamah, Ranjana Mehta, Farzan Sasangohar

**Affiliations:** 1 Department of Industrial and Systems Engineering Texas A&M University College Station, TX United States; 2 Center for Outcomes Research Houston Methodist Hospital Houston, TX United States

**Keywords:** mHealth, apps, wearable sensors, substance abuse disorder, mobile phone

## Abstract

**Background:**

Advances in technology engender the investigation of technological solutions to opioid use disorder (OUD). However, in comparison to chronic disease management, the application of mobile health (mHealth) to OUD has been limited.

**Objective:**

The overarching aim of our research was to design OUD management technologies that utilize wearable sensors to provide continuous monitoring capabilities. The objectives of this study were to (1) document the currently available opioid-related mHealth apps, (2) review past and existing technology solutions that address OUD, and (3) discuss opportunities for technological withdrawal management solutions.

**Methods:**

We used a two-phase parallel search approach: (1) an app search to determine the availability of opioid-related mHealth apps and (2) a scoping review of relevant literature to identify relevant technologies and mHealth apps used to address OUD.

**Results:**

The app search revealed a steady rise in app development, with most apps being clinician-facing. Most of the apps were designed to aid in opioid dose conversion. Despite the availability of these apps, the scoping review found no study that investigated the efficacy of mHealth apps to address OUD.

**Conclusions:**

Our findings highlight a general gap in technological solutions of OUD management and the potential for mHealth apps and wearable sensors to address OUD.

## Introduction

### Background

On average, 5 people in the United States die every hour from an opioid overdose [1]. In 2017 alone, over 70,000 deaths occurred due to drug overdose [2]. This problematic pattern of opioid use often referred to as opioid use disorder (OUD), is considered a public health emergency [1,3] with significant negative impacts on health care [4,5] and criminal justice costs [6]. Misuse of opioids can occur among patients who are initially exposed to opioids in the perioperative period—periods immediately before, during, and after a surgical operation—or through a prescription for the treatment of acute or chronic pain [7]. In addition, opioids attract illegal users and individuals who profit by selling them unlawfully [8]. Such illegitimate use of prescription opioids has exacerbated the increase in OUDs [9-11].

Treatment exists for OUD, comprising pharmacotherapy and behavioral therapies [12,13]. Opioid-dependent users may experience challenging and often severe withdrawal symptoms, including restlessness, muscle aches, and depression, when they abruptly discontinue or reduce opioid intake [14]. Irrespective of the OUD treatment path, opioid withdrawal management, which includes regularly monitoring patients for symptoms, is the crucial first step after opioid use cessation or dose reduction [1]. A review of opioid withdrawal monitoring methods [15] revealed that the current method of assessing opioid withdrawal using various scales (tools to monitor and rate common signs and symptoms of withdrawal) is self-reported, requires frequent observations, may suffer from recall bias (a study by Infante-Rivard and Jacques [16] gives more details)—unintentional or intentional underreporting of information by respondents—and is ineffective outside of clinic or research environments. Moreover, opioid withdrawal scales differ with regard to the number of scale items and rating criteria. Although technologies such as electronic prescription systems for controlled substances [17], medication history repositories, exchange of clinical records, and clinical direct messaging [8] have been proposed as useful methods to address opioid management, an opioid monitoring method that noninvasively and continuously monitors patients’ symptoms as they occur in real time would provide several distinct advantages over the existing methods [18].

Advancements in technology have allowed the continuous monitoring of diseases outside of clinical settings. Mobile health (mHealth), one such advancement, involves the use of mobile devices to collect health data, monitor signs and symptoms, deliver remote care, and/or educate patients [19]. mHealth interventions allow medical content to be delivered anytime and anywhere to patients [20]. mHealth apps have been used to manage chronic diseases, including monitoring and managing day-to-day symptoms of sickle cell disease [21,22]; monitoring patients undergoing cardiac rehabilitation [23]; monitoring blood pressure measurements to control hypertension [24]; monitoring blood glucose, blood pressure, and physical activity to prevent metabolic syndrome [25]; and monitoring patients with chronic obstructive pulmonary disease [26] (a study by Hamine et al [27] gives a systematic review of mHealth apps for chronic disease management). However, in comparison to chronic disease management, the application of mHealth to OUD has been limited. Digital health technologies, including mHealth apps, have the potential to play a unique role in tackling OUD. These include enabling care providers to create digital profiles of patients to provide personalized care regardless of time and place, monitoring patients’ vital trends and issuing alerts to them or their caregivers, and providing insights into what triggers patients’ behaviors.

### Objectives

Inspired by this gap, the overarching aim of our research was to design OUD management technologies that utilize wearable sensors to provide continuous monitoring capabilities. In particular, this research addresses the missed opportunity in monitoring withdrawal symptoms, given their acute nature, salient physiological correlates, and their importance to long-term sobriety. As the first step in investigating novel technological solutions for remote monitoring and management of OUD and, in particular, withdrawal symptoms, we investigated the availability and evidence to support the efficacy of current mHealth and wearable sensor solutions for OUD. The objectives of this paper were to (1) document the currently available opioid-related mHealth apps, (2) review the past and existing technology solutions that address OUD, and (3) to discuss opportunities for technological withdrawal management solutions. To the best of our knowledge, no such review or landscape analysis of technologies that address OUD has been conducted to date.

## Methods

### Overview

A two-phase parallel search approach was used, which involved an app search to determine the availability of opioid-related mHealth apps, and a scoping review of relevant literature was undertaken to identify relevant technologies and mHealth apps used to address OUD. The Preferred Reporting Items for Systematic Reviews and Meta-Analyses Extension for Scoping Reviews guidelines were used [28].

### Mobile Health App Search Method

A search was conducted on the Apple App Store and Google Play for apps published until May 10, 2019, using a combination of search terms that included “opioid”, “opiate”, “substance use disorder”, “technology”, OR “addiction”. The inclusion criteria were as follows: relevance to opioid, opioid prescription, opioid training, opioid monitoring, opioid overdose, opioid addiction support, or substance use disorder (SUD), including opioids. Apps that used a non-English language, apps that solely addressed SUD but not specific to opioids, and apps that required a web browser to use were excluded.

Overall, 2 reviewers independently applied the inclusion and exclusion criteria and identified the final set of apps for review. For each app, the reviewers independently extracted the following: app name, app description, year published, publisher or seller, download estimate, rating, and price. The reviewers transferred the extracted data to a detailed Excel spreadsheet. Then, the reviewers coded the apps for the operating system, that is, Android operating system (henceforth Android) and iPhone operating system (henceforth iOS); clinical focus (opioid-specific or SUD including opioid); audience (patients, clinicians, or anyone); and function (medication-assisted treatment, education, prescription, professional support, peer support, withdrawal support, and patient monitoring; Table 1). Each app was assigned to one primary audience and clinical focus; however, each app could be categorized under more than one app function. Disagreements regarding exclusion/inclusion and coding of the apps were discussed with a third reviewer, and agreement was reached through discussion.

### Scoping Review Method

PubMed, Excerpta Medica Database (EMBASE), and Google Scholar were searched for articles published from their inception until May 10, 2019, using a combination of search terms: (“wearable” OR “sensors” OR “technology” OR “mHealth” OR “app” OR “mobile”) AND (“opioid use disorder” OR “opioid” OR “opiate”). Studies were included if they (1) were in English, (2) were peer reviewed, and (3) employed wearable sensors and/or mHealth. Animal studies and studies that did not include opioids were excluded.

**Figure 1:**
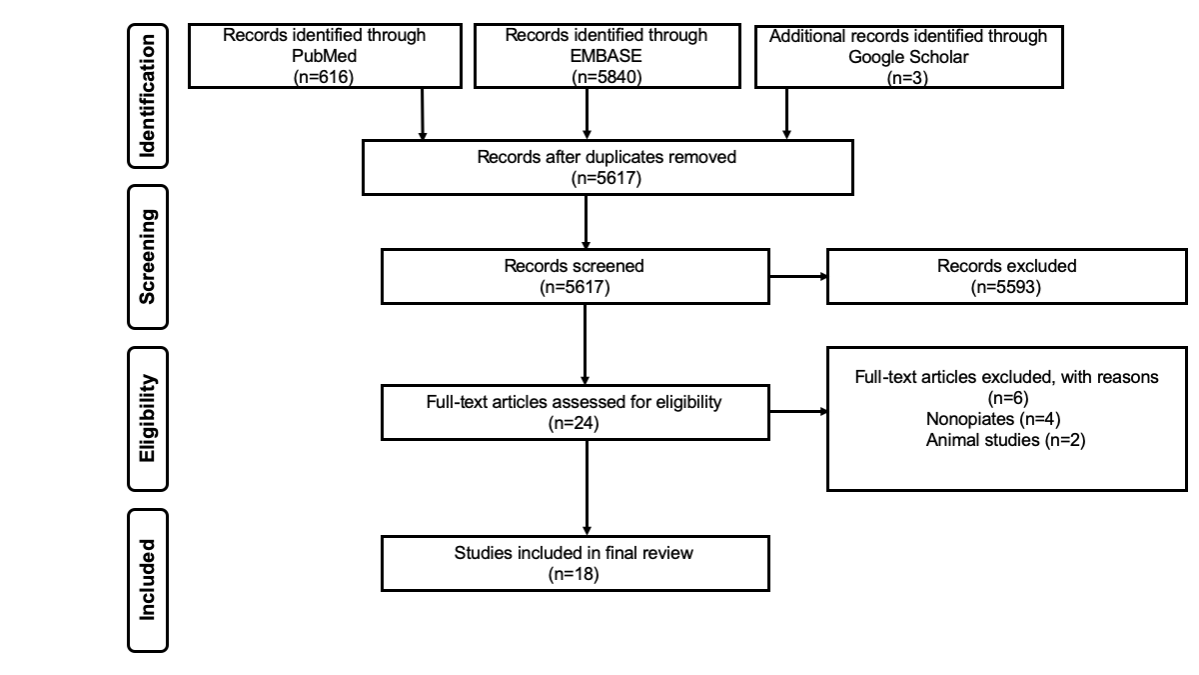
Preferred Reporting Items for Systematic Reviews and Meta-Analyses (PRISMA) diagram showing the process of searching and selecting studies included in the review. EMBASE: Excerpta Medica Database.

The selection of articles was conducted in 2 stages. In the first stage, 2 reviewers independently reviewed titles and abstracts against the inclusion and exclusion criteria using a web-based tool for systematic and scoping reviews called Rayyan [29]. The decision to fully review an article was made when both reviewers agreed to include the abstract. The reviewers resolved disagreements regarding article eligibility by discussing with a third reviewer. In the second stage, full-text articles were reviewed to determine eligibility. Furthermore, backward and forward reference searches were conducted on all full-text articles that met the study selection criteria. Figure 1 shows the process of searching and selecting articles included in the review. Secondary searching yielded no unique results.

**Table 1:** Taxonomy used for mobile health app coding.

Code and category		Description
**Audience**		
	Patient-facing	App supports patient interactions and engagement
	Clinician-facing	App assists physician decision making
	Anyone	App that is designed for general public, including patients and caregivers
**Clinical focus**		
	Opioid-specific	App related to only opioids
	Substance use disorder	App related to substances, including opioids
**App function**		
	Medication-assisted treatment	App supports medication-assisted treatment of opioid use disorder
	Education	App provides educational information
	Conversion	App helps generate equivalent doses of various oral and intravenous opioids
	Professional support	App provides connections to outside professional support, eg, sends a message through the app to seek immediate emergency assistance, finds services and resources that are available nearby
	Peer support	App provides connections to peer support, including individuals undergoing rehabilitation
	Withdrawal support	App supports patients as they go through withdrawal with, eg, reminders, supportive messages, symptom library
	Patient monitoring	App prompts patients to self-evaluate and submit regular personal assessments directly for the purpose of tracking progress and patterns of behavior

Furthermore, 2 reviewers independently read the full text of each article identified for inclusion in the review to extract pertinent data using a data extraction form. From each article, the reviewers independently extracted the following: technologies used, physiological parameters, functions, research methods employed, and study findings. The reviewers transferred the extracted data to a detailed Excel spreadsheet. The technologies used were further organized into ecological momentary assessment (EMA), GPS information, wearable sensors, machine learning, and biomedical devices.

## Results

### Mobile Health App Search Results

**Figure 2:**
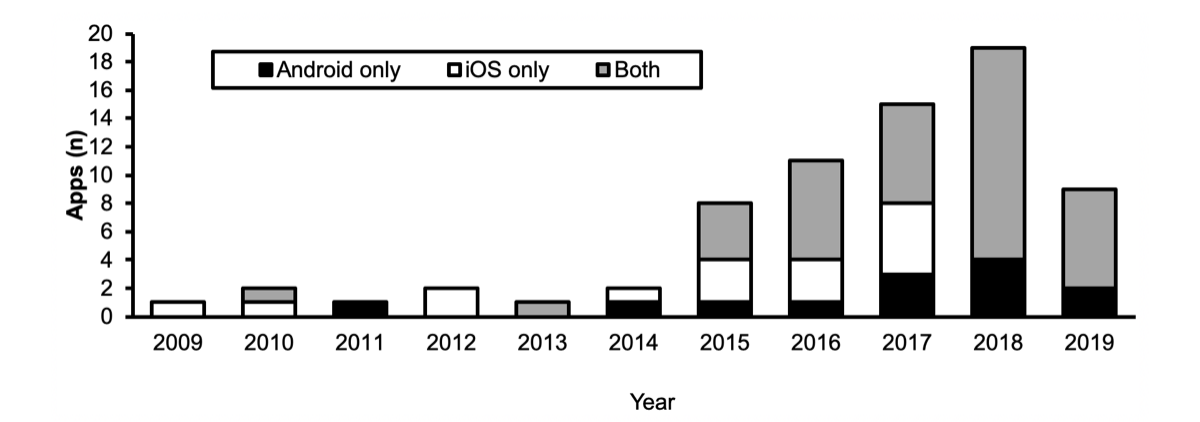
Graph showing the number of apps published from January 2009 to May 10, 2019. iOS: iPhone operating system.

The search yielded a total of 72 apps. Of the 72 apps, 62 apps (86%) were available for download at no cost. The remaining 10 (10/72, 12%), all clinician-facing apps, had prices ranging from US $0.99 to US $9.99. Figure 2 shows the number of apps that were made available from January 2009 to May 10, 2019, for both operating systems. Table 2 shows the apps categorized by the audience and the operating system. Clinician-facing apps were most frequently available (31/72, 43%), followed by apps that could be used by patients, caregivers, or the general public (23/72, 32%). As shown in Table 3, most of the available apps were opioid-specific (62/72, 86%).

**Table 2:** Apps categorized by audience and operating system.

Operating system	Apps categorized by audience, n (%)			Total apps, n
	Patient-facing	Clinician-facing	General audience	
Android only	3 (23)	8 (61)	2 (15)	13
iOS^a^ only	1 (5)	14 (82)	2 (11)	17
Both Android and iOS	14 (33)	9 (21)	19 (45)	42
Total	18 (25)	31 (43)	23 (32)	72

^a^iOS: iPhone operating system.

**Table 3:** Apps categorized by clinical focus and operating system.

Operating system	Apps categorized by clinical focus, n (%)		Total apps, n
	Opioid-specific	Substance use disorder	
Android only	11 (84)	2 (15)	13
iOS^a^ only	15 (88)	2 (11)	17
Both Android and iOS	36 (85)	6 (14)	42
Total	62 (86)	10 (13)	72

^a^iOS: iPhone operating system.

Furthermore, apps were analyzed for utilities (Table 4). Although most apps provided opioid conversion support (25/72,35%) or educational content (21/72, 29%), only 2 opioid-specific apps (2/62, 3%), namely, FlexDek for medication-assisted treatment (MAT) and MATx by Substance Abuse and Mental Health Services Administration (SAMHSA), were designed to support medication-assisted treatment and 4 apps (4/72, 5%) provided support for patient monitoring.

**Figure 3:**
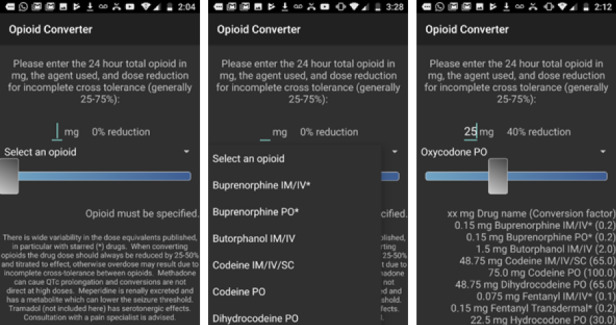
Screenshots of Opioid Converter app showing the main interface (left), selection of an opioid (center), and 25 mg oxycodone adjusted at 40% for incomplete cross-tolerance (right).

Most apps (25/72, 35%), all clinician-facing and opioid-specific, were developed to convert from one opioid to another. These were also the most downloaded apps (Table 5). For example, Opioid Converter (Figure 3), the app with the highest number of downloads, is a free app supported by Emory University and is designed to aid with opioid dose conversions. The app has a slider that allows for adjustments to be made for incomplete cross-tolerance. The opioids covered include buprenorphine, butorphanol, codeine, fentanyl, hydrocodone, morphine, and oxycodone.

**Table 4:** App tallies for different function categories (utilities are not mutually exclusive).

Clinical focus	App categorized as per their functionality, n (%)							
	Medication-assisted treatment	Education	Converter	Professional support	Peer support	Withdrawal support	Patient monitoring	Other
Opioid-specific (n=62)	2 (3)	16 (25)	25 (40)	8 (12)	4 (6)	2 (3)	4 (6)	1 (1)
Substance use disorder (n=10)	1 (10)	5 (50)	0 (0)	1 (10)	2 (20)	0 (0)	0 (0)	1 (10)
Total (n=72)	3 (4)	21 (29)	25 (35)	9 (12)	6 (8)	2 (2)	4 (5)	2 (2)

**Table 5:** Most downloaded Android apps.

App name	Year published	Rating (out of 5)	Reviews, n	Estimated downloads, n
Opioid Converter	2011	4.0	170	50,000+
Orthodose	2013	4.6	56	10,000+
Opioid Calculator	2016	4.0	34	10,000+
CDC^a^ Opioid Guideline	2016	2.8	17	10,000+
Painkiller Calculator	2014	4.2	21	5000+
FEND by Preventum	2018	4.2	32	5000+

^a^CDC: Centers for Disease Control and Prevention.

Overall, 9 of the 72 apps (9/72, 12%) were designed to provide professional support, including connecting users with a network of service providers and finding naloxone carriers in an overdose emergency. Furthermore, 6 of the 72 apps (6/72, 8%) were designed to provide peer support in the form of reminders, supportive messages, and symptom library. In addition, 4 out of 72 apps (4/72, 6%) were designed to provide patient monitoring by prompting patients to self-evaluate and submit regular personal assessments directly for the purpose of tracking progress and patterns of behavior. Overall 2 out of 72 apps (2/72, 3%) were categorized as *other*. One of these apps, Diagnosis, Intractability, Risk, and Efficacy (DIRE), is designed for clinicians to use DIRE as a tool [30] in their decision-making process when considering prescribing opioids. The DIRE tool allows clinicians to rate 7 factors (diagnosis, intractability, psychological risk, chemical health risk, reliability risk, social support risk, and efficacy) on a scale of 1 to 3, with 1 being the least favorable case for prescribing opioids and 3 being the most favorable case for prescribing opioids. The total score (ie, the sum of the ratings) is used to determine a patient’s suitability for opioid maintenance analgesia. The other app is THRIVEE, a virtual platform system designed to help patients overcome addiction. THRIVEE delivers virtual MAT to addicts, including opioid users. It utilizes virtual telemedicine sessions such as video conferencing between patients and providers to leverage proven clinical practices.

**Figure 4:**
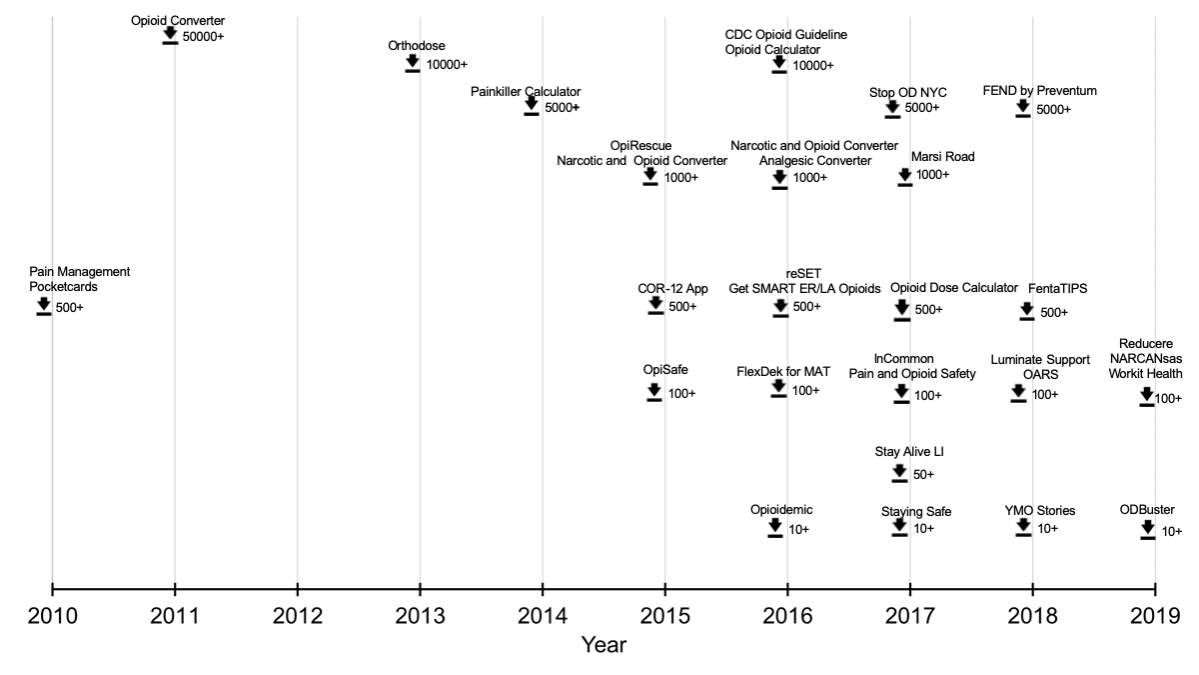
Timeline of the apps showing the year each app was first published (on the horizontal axis) versus the estimated number of downloads from the date the app was published to the search date (on the vertical axis). Timeline for most downloaded Android apps showing the number of downloads from January 2010 to May 10, 2019. Download statistics are not available for iPhone operating system–based apps. FEND: Full Energy No Drugs. CDC: Centers for Disease Control and Prevention. OARS: Opioid Addiction Recovery Support. MAT: Medication Assisted Treatment.

The total number of downloads was used as a measure of app prevalence. Although the download statistics were not available for iOS apps, the statistics for Android apps varied from as low as 5+ downloads to as high as 50,000+ downloads (Figure 4). Table 5 shows the 6 most downloaded Android apps and their respective ratings.

### Focused Review Results

Our initial search yielded 6459 articles. These were exported to the Zotero reference management software, where 842 duplicates were removed. Title and abstract screening resulted in the exclusion of 5593 articles. The remaining 24 articles were fully reviewed. Of these 24 articles, 18 met the inclusion criteria and were included in the final review.

Our search yielded 18 papers that documented relevant technologies used to address OUD. Of the 18 studies, 9 (50%) were laboratory-based studies, 8 (44%) were field studies, and 1 (6%) was a clinical trial. We did not find studies that employed mHealth apps to address OUD. Table 6 presents a summary of the technologies identified in the scoping review.

**Table 6:** Technologies identified in the scoping review.

Article	Technologies	Physiological parameters	Utility	Methods
Epstein et al [31]	PDA^a^ (Palm Zire, PZ21) and diary software	N/A^b^	Monitoring	5 random prompts per day (5 weeks) and 2 random prompts per day (20 weeks)
Boyer et al [32]	Smartphones, wearable sensors, and machine learning	EDA^c^, acceleration, skin temperature, and heart rate	Real-time detection of drug craving and interventions	Self-annotation of physiological changes and machine learning
Epstein and Preston [33]	PDA (Palm Zire, Palm Zire 21) and diary software	N/A	Momentary ratings of stress in outpatients at work	5 random prompts per day (5 weeks) and 2 random prompts per day (20 weeks)
Kennedy et al [34]	PDA (Palm Zire, PZ21) and diary software	N/A	Gender-based treatment strategies	Random prompts (2-5 per day) for location, activities, and companions
Epstein et al [35]	PDA (PalmPilot) and GPS (BT-Q1000X)	N/A	Real-time monitoring of mood, stress, and drug craving	Time-stamped GPS data and EMA^d^ ratings of mood, stress, and drug craving
Kennedy et al [36]	Biosensor (AutoSense) and smartphone	Heart rate	Continuous monitoring of heart rate	Wireless heart rate sensor data and self-reports
Carreiro et al [37]	Biosensor (Q sensor)	EDA, skin temperature, and acceleration	Real-time detection of drug use	Continuous monitoring of EDA, skin temperature, and acceleration
Linas et al [38]	PharmChek drugs of abuse patches, Palm Z22, and smartphone	Sweat patches detect traces of cocaine or heroin secreted in sweat during the period they are worn	Agreement of EMA methods with other methods (ie, biological and ACASI^e)^ of assessing drug use	Palm Z22 PDA (3 trials) and Motorola Droid X2 phone (1 trial), self-reports of heroin or cocaine, sweat patches (weekly), and ACASI (weekly)
Mennis et al [39]	Smartphone and GPS	N/A	Integration of GPS information with EMA to study neighborhood effects on opioid use disorder	Combined GPS information with EMA to find association among neighborhood disadvantage, perceived stress, perceived safety, and substance use; generalized estimated equations for analysis
Sarker et al [40]	Biosensor, smartphone, GPS, and machine learning	ECG^f^ and inspiratory to expiratory ratio	Time series health data to determine the timing of interventions and links to prevent drug craving and relapse	Smartphone initiated–32-item EMA (random); modeling R-R intervals and heart rate variability from ECG data
Carreiro et al [18]	Biosensor (Q sensor)	EDA, skin temperature, and acceleration	Biosensors may be used in drug addiction treatment and pain management	Hilbert transform analyses combined with paired *t* tests to compare biosensor data
Wang et al [41]	Biosensor (Q sensor), urine drug screens, and patient self-report of substance use	EDA, skin temperature, and acceleration	Detect and set up thresholds of parameters in real-time drug use event detection for wearable biosensor data streams	Sliding window technique to process data stream and distance-based outlier algorithm to detect substance use events
Chintha et al [42]	Biosensor (Empatica E4)	Skin temperature, acceleration, and heart rate	Identify physiologic change that marks wearing off of naloxone effect	90-min postnaloxone time point evaluated with Hilbert transform
Kowalczyk et al [43]	PalmOne Zire 21, Palm Tungsten E2, or HTC TyTN II smartphone	N/A	Investigate the relationship between opioid use and craving and affect	Mobile devices used to rate craving 4 times randomly each day
Mahmud et al [44]	Biosensor (Q sensor) and machine learning	EDA and skin temperature	Automatic detection of opioid intake and classification of pre- and postopioid health conditions	Time and frequency domain feature analysis; decision tree, k-nearest neighbors, and extreme gradient boosting classifiers
Moran et al [45]	Smartphone	N/A	Gender differences in the influence of stress on opioid use and craving	Entry was initiated, and causes, context, stress, and craving severity were rated each time the participant felt more stressed than usual
Preston et al [46]	Smartphone	N/A	Relationship between daily hassles and stressful events in opioid-dependent men and women	Randomly prompted entries, self-initiated reports of drug use, self-initiated reports of stressful events, and end-of-day entries
Miranda and Taca [47]	BRIDGE—an auricular neurostimulation device	Not reported	Treat opioid withdrawal symptoms without the use of antiopioids	Patients wore device behind the ear to stimulate nerves in brain and spinal cord

^a^PDA: personal digital assistant.

^b^N/A: not applicable.

^c^EDA: electrodermal activity.

^d^EMA: ecological momentary assessment.

^e^ACASI: audio computer-assisted self-interviewing.

^f^ECG: electrocardiogram.

#### Ecological Momentary Assessment

Overall, 6 studies (6/18, 33%), all field-based, employed EMA, a method that uses electronic diaries and/or questionnaires deployed on mobile devices [48] to monitor, in near real time, the craving for and use of opioids by outpatients receiving methadone treatment [31]; assess stress in outpatients at work [33]; investigate gender-based treatment strategies [34]; study the relationship between opioid use and craving and affect [43]; investigate gender differences in the influence of stress on opioid use and craving [45]; and examine the relationship between daily hassles and stressful events in opioid-dependent men and women [46]. Epstein and Preston [33] found opioid-dependent outpatients to be less stressed at the workplace than elsewhere, demonstrating the utility of EMA to rate stress in outpatients. Kennedy et al [34] found that males and females with SUD differ in their daily functioning during addiction treatment, highlighting the need to develop gender-based treatment strategies. Similarly, Moran et al [45] found that stress-induced craving differs between opioid-dependent men and women, suggesting that gender-based tailoring of treatment should consider individual differences. Kowalczyk et al [43] found that cravings increased when the participants were using opioids, indicating the utility of EMA to investigate the relationship between opioid use and craving. Overall, EMA has shown promise in enabling the measurement of momentary experiences and states of cravings and misuse in natural settings.

#### GPS Information

Overall, 2 studies (2/18, 11%), both field-based, combined EMA with GPS location information to monitor the real-time mood, stress, and drug craving in a geographical context [35] and to study neighborhood effects on substance use [49]. EMA provided the participants’ momentary experience, whereas GPS provided the participants’ location during those experiences. Epstein et al [35] found a negative association among environmental disorders (defined as a lack of order and social control within the neighborhood) [49] and mood, stress, and drug craving, suggesting that mood, stress, and drug craving can be monitored in real time in a geographical context. Mennis et al [39] found a significant positive association among neighborhood disadvantage, higher perceived stress, lower perceived safety, and greater substance use, suggesting that GPS information can be combined with EMA to study neighborhood effects on substance use.

#### Wearable Sensors

The advances in wearable technologies have enhanced the ability of researchers to monitor physiological changes associated with opioid intake and/or drug craving. Overall, 8 out of the 18 studies (8/18, 44%) employed wearable sensors. Of these 8 studies, 3 (37%) studies [32,36,40] combined EMA and wearable sensors to detect drug cravings [32,36], deliver personalized prevention interventions [32], and determine stress episodes among opioid users [40]. Kennedy et al [36] reported higher heart rates when participants reported craving compared with when they reported no craving, suggesting the potential efficacy of using heart rate data for continuous monitoring of craving. The *iHeal* system [32]—a system architecture intended to provide personalized interventions—combines EMA, wearable sensors, and a deep belief network model to detect drug cravings and deliver personalized drug prevention interventions. However, this study did not implement their *iHeal* system.

The remaining 5 (5/8, 63%) studies [18,37,41,42,44] used wearable biosensors for real-time detection of opioid use [37,41] to detect physiological changes associated with opioid use [18], evaluate physiological changes associated with the wearing off of naloxone [42], and automatically detect opioid intake [44]. Studies using Q sensors, worn on the participants’ wrists, have found that an increase in electrodermal activity (EDA) is associated with opioid use [41], accurately detected substance use events within 30 min [41] and significant within-subjects increase in skin temperature and decrease in locomotion immediately after opioid administration [18]. Carreiro et al [18] found that physiological changes varied among subjects with the levels of opioid use—heavy opioid users showed a greater decrease in fidgeting movements than nonheavy opioid users. Chintha et al [42] used an E4 device (Empatica) worn on the participants’ wrists and found that heart rate and skin temperature differed significantly between before and after naloxone administration. Finally, Linas et al [38] combined EMA and wearable sweat patches, PharmChek Drugs of Abuse Patches (PharmChem Inc), to concurrently collect momentary data and sweat in the field from 109 adults with recent opioid use and found moderate-to-good agreement of EMA to sweat patches and self-report methods in capturing drug use events.

#### Machine Learning

Overall, 4 studies (4/18, 22%) used machine learning techniques to analyze and predict opioid use. Furthermore, 3 (3/18, 17%) of these studies [40,41,44] predicted opioid intake. The remaining study [39] developed a model to provide personalized interventions. Sarker et al [40] combined EMA; location information; the cStress model (reported on in a study by Hovsepian et al [50]), which uses electrocardiogram (ECG) and respiration data; and the moving average convergence divergence method to predict stress episodes associated with opioid intake. Their model predicted stress episodes with an accuracy of 94.8% and kappa of 0.444. Wang et al [41] used a sliding window technique to process streams of EDA, skin temperature, and acceleration data collected from a wrist-worn Q sensor and a distance-based outlier algorithm to detect substance use events. Their model accurately detected substance use events within 30 min. Using 2 parameters, movement in the z-axis and skin temperature collected from wrist-worn Q sensors, Mahmud et al [44] compared the ability of 3 classifiers (decision tree, k-nearest neighbors, and extreme gradient boosting) to automatically detect opioid intake, obtaining an accuracy of 99.4% with extreme gradient boosting.

#### Biomedical Devices

Miranda and Taca [47] investigated the effect of an auricular neurostimulation device, BRIDGE, in treating opioid withdrawal symptoms. The device was placed behind the ears of 73 opioid-dependent outpatients for a maximum of 5 days to treat opioid withdrawal symptoms by stimulating the nerves in the brain and spinal cord. A reduction in opioid withdrawal scores, measured with the clinical opioid withdrawal scale, was associated with the use of BRIDGE.

## Discussion

### Principal Findings

The goal of the app search in this study was to determine the availability of opioid-related mHealth apps. The search revealed the availability of 72 Android and iOS apps and revealed a steady rise in app development from January 2009 to May 10, 2019, with most apps designed to support clinicians. Our findings suggest that most of the apps have been developed to help clinicians convert from one opioid to another at an equianalgesic dose. Despite the availability of these apps, the scoping review found no study that investigated the efficacy of mHealth apps to address OUD.

### Outcome Evaluation of Mobile Health App Search

Opioid conversion, a common but challenging clinical practice [51], is required when patients do not respond therapeutically, develop adverse effects to an opioid, or need an alternative route of administration [52]. Prescription error has been identified as a significant risk factor for opioid-related deaths [53]; therefore, opioid conversion apps that run on mobile devices may help improve patient safety [54]. Although these apps are not geared toward OUD, they help primary care providers safely prescribe opioids.

The US Food and Drug Administration (FDA) has the mandate to regulate mHealth apps that meet certain statutory criteria as medical devices. Under the existing FDA regulatory framework, it is difficult to determine whether an mHealth app is a medical device [55]. The FDA has long exempted apps considered as *low-risk* from its approval process [56]. It is unclear how many opioid conversion apps identified in this study have been approved by the FDA. For example, although Pear reSET-O, a prescription app, was first published in 2016, it is only recently that the FDA cleared it as the first software-generated therapeutic intervention for patients with OUD [57]. This app provides cognitive behavioral therapy to patients enrolled in an OUD treatment program.

Although most apps identified in this study are free to download, many health care providers and patients may not be aware of their availability. Future studies should investigate such awareness and adoption rates. The factors that influence the adoption of mHealth apps by health professionals include lack of clinical evidence [58], security [59], and inability to integrate apps with other systems [60]. The factors that influence patients’ adoption of mHealth apps include security and privacy concerns [61,62], social contacts [63], and cost of smartphones and data plans [62,64]. Failure to balance system demands of apps with end user needs and resources undermines the adoption of mHealth apps [65]. Conducting content analyses, usability testing, observational studies, and efficacy testing will contribute to the increased adoption of mHealth apps in clinical practice [66].

#### Privacy of Mobile Health Apps

The privacy of mHealth apps, the right for users to know how their information is collected and used, is an issue worthy of discussion. In this study, most apps identified in the search were free to download. For users of these apps, there is a likelihood that their information is passed around to third parties, thereby exposing them to privacy risks [67]. A recent study investigated data sharing practices in the mHealth ecosystem and found that 79% of the sampled apps shared user data with 55 entities, including third parties [68]. This presents a major concern for mHealth users as they do not know how their data will be used and by whom. Furthermore, the aggressive medicolegal system in the United States deters many health care providers from using mHealth apps. Recent studies (eg, study by Hutton et al [69]) have suggested the need for standards that can ensure mHealth app user privacy.

### Outcome Evaluation of Focused Review

Despite the availability of opioid-related apps, this scoping review, which sought to document relevant technology solutions that address OUD, found no study that employed mHealth apps to address OUD. Most of the studies employed EMA to capture participants’ opioid use patterns as they occurred in real time. Few studies have combined EMA with a range of data types, including physiological changes and location information, to detect opioid intake. These findings highlight a general lack of empirical evidence to support the efficacy of mHealth apps for OUD management. However, our findings show the potential for wearable sensors, especially in opioid withdrawal management, to facilitate remote monitoring of the signs and symptoms of OUD.

Opioid withdrawal management, which includes regular monitoring of patients for symptoms, is the crucial first step after opioid use cessation or dose reduction [1]. The relapse rates during inpatient treatment of opioid dependence indicate that as many as 91% of those in recovery experience an opiate relapse, 59% of whom relapse within the first week of sobriety, and 80% within a month after discharge from a detox program [70]. The results from this scoping review revealed that most of the studies employed EMA or combined EMA with a range of data types to detect opioid usage patterns. These studies focused on opioid intake and usage patterns. Only one study [47] focused on developing technology to help treat opioid withdrawal symptoms. Indeed, the BRIDGE device used in that study is the first of its kind approved by the FDA. It is crucial that technology solutions be provided not only to help health care professionals monitor and manage patients’ withdrawal symptoms but also to help the patients themselves as they go through withdrawal.

### Gaps Identified in Outcome Evaluation of Technologies for Opioid Use Disorder Management

From the results of this study, it is evident that there is a gap in the technologies available to manage opioid withdrawal. Advances in wearable and machine learning technologies have enhanced the ability of researchers to monitor physiological changes associated with opioid intake and/or drug craving [40,41,44]. In the same vein, wearable sensors can be employed to detect temporal and spectral patterns of physiological responses associated with opioid withdrawal symptoms. For example, joint/muscle aches lead to elevated heart rate [71], which can be measured with a wearable ECG; anxiety leads to elevated heart rate [72] and change in skin conductance [73], which can be measured using wearable ECG and EDA sensors, respectively; and *cutis anserine,* defined as goosebumps, leads to a change in skin conductance [74], which can be measured with a wearable EDA sensor. Machine learning–based pattern detection algorithms may be used to explicitly detect and characterize specific features obtained from wearable sensor configurations and existing contextual information. This can provide real-time feedback to health care providers to facilitate interventions.

### Limitations

There are some limitations in the study that warrant discussion. First, the search may not be collectively exhaustive because of the limitations of the scoping review. The scoping review utilized relatively fewer, albeit relevant, number of search terms and databases to identify potentially eligible studies. Despite this limitation, we found saturation in the technologies used to address OUD, evidenced by the lack of additional results from the 19-article–based bibliographic secondary search. Second, the availability of information about app downloads was limited to Android apps only. However, the data presented in this study are relevant, given that Android has overtaken iOS as the number 1 operating system for mHealth apps [75]. Third, although the app rating information is reported, it is difficult to determine how many of the ratings were legitimately written by people who used the apps. In addition, we were unable to determine how the apps were rated. Owing to this lack of information, this study did not include information on the quality of the apps. Furthermore, we did not focus on capturing the apps’ effectiveness. Given the proliferation of mHealth apps and technologies made available to target OUD, future studies should aim to investigate the quality and effectiveness of these apps on OUD management. Finally, developers may be reluctant to publish research on their apps for intellectual property reasons (if they have any); many of their results/algorithms may be considered *proprietary*.

### Conclusions

This study showed the availability of opioid-relevant mHealth apps, most of which are opioid conversion apps. Despite the availability of these apps, the scoping review found no study that employed mHealth apps to address OUD. Most studies employed EMA to capture the participants’ opioid usage patterns as they occurred in real time. Few studies have combined EMA with a range of data types, including physiological changes and location information, to detect opioid intake. Our findings highlight the gap in technologies and the potential for using wearable sensors, especially in opioid withdrawal management, to address OUD.

## Abbreviations

DIRE: Diagnosis, Intractability, Risk, and Efficacy
ECG: electrocardiogram
EDA: electrodermal activity
EMA: ecological momentary assessment
FDA: Food and Drug Administration
iOS: iPhone operating system
mHealth: mobile health
OUD: opioid use disorder
SUD: substance use disorder
